# Comparison of traffic collision victims between older and younger drivers in South Korea: Epidemiologic characteristics, risk factors and types of collisions

**DOI:** 10.1371/journal.pone.0214205

**Published:** 2019-04-09

**Authors:** Jae Yun Ahn, Hyun Wook Ryoo, Jung Bae Park, Jong Kun Kim, Mi Jin Lee, Dong Eun Lee, Kang Suk Seo, Yun Jeong Kim, Sungbae Moon

**Affiliations:** 1 Department of Emergency Medicine, School of Medicine, Kyungpook National University, Daegu, South Korea; 2 Department of Emergency Medicine, School of Medicine, Kyungpook National University, Kyungpook National University Hospital, Daegu, South Korea; University of Toronto, CANADA

## Abstract

**Objective:**

This study aimed to show the epidemiological characteristics and the difference in the risk factors and types of collision between older and younger drivers in Korea.

**Methods:**

We collected data from the Emergency Department-based Injury In-depth Surveillance retrieved by the Korea Centers for Disease Control and Prevention from 2011 to 2015. We included injured drivers aged ≥ 18 years who were registered in the database, who were limited to drivers of four-wheeled vehicles. The enrolled patients were divided according to age into older (≥ 65 years) and younger (< 65 years) drivers. The total number of enrolled drivers was 37,511; 2,361 (6.3%) of them were older drivers. The epidemiological characteristics (e.g., age, sex, fatality rate) of traffic collision victims for 5 years were determined, and the risk factors (e.g., seat belt use) and types of collision (single- vs. multi-vehicle) between the two groups were compared.

**Results:**

The median age and interquartile range (IQR; 25^th^ and 75^th^ percentiles) of all drivers were 41.0 (IQR, 32.0–52.0), and 24,544 (65.4%) of them were men. The median age increased from 40.0 (IQR, 31.0–50.0) to 43.0 (IQR, 33.0–54.0) between 2010 and 2015 (*P* < 0.001). The proportion of older drivers increased from 5.0% to 8.4% annually during the study period (*P* < 0.001). Between 2010 and 2015, the fatality rate decreased from 3.1% to 1.2% (*P* = 0.287) for older drivers and from 0.9% to 0.5% (*P* = 0.009) for younger drivers. The proportion of single-vehicle collision (25.9% vs. 20.3%) was higher in older than in younger drivers (*P* < 0.001). Older drivers had a lower rate of seat-belt use than younger drivers (79.0% vs. 83.0%, *P* < 0.001).

**Conclusions:**

The proportion of older drivers increased annually during the study period, and older drivers experienced more single-vehicle collision and used seat belt less frequently than younger drivers. A national policy support to reduce traffic collision in older drivers and public relation activities to enhance their seat belt use should be strengthened in the future.

## Introduction

South Korea has a rapidly aging population. In 2015, 13.2% of South Korea’s population was determined to be ≥ 65 years of age [1]. With the increase in elderly citizens, the proportion of older adults with driver’s licenses in South Korea has also increased, from 7.6% in 2013 to 9.6% in 2016 [2]. Hanrahan et al. estimated that the number of drivers aged ≥ 65 years will double in 2030 compared to 2010 and 25% of all fatal crash involvements will involve older drivers in 2030 in the United States [3]. Thompson reported that the rates of crashes and serious or fatal injuries per 100,000 population and 100,000 licensed drivers for all age groups in Australia had decreased between 2002 and 2013, except those aged 85 years, which remained constant or increased; therefore, more research attention is required to improve their road safety [4].

A variety of conceptual models for injury prevention have been proposed, among which the Haddon matrix is a representative tool that enables analysis of multiple factors associated with traffic collisions, and consists of the risk factors for human, vehicles, and environment at each phase (before, during, and after the crash) [5–7]. Among these factors, human-related factors include vehicle speed, driving skill of the driver, physical disability at pre-crash phase, wearing a seat belt or equipped with an airbag during the crash phase, and health status of the victim, first-aid skill, access to medic after the crash phase. To know the difference in the characteristics between the older and younger driver traffic collision in human-related factors are important to establish policies for preventing injury and reducing the severity in the event of a collision by older drivers. Generally, young drivers are more likely to be involved in collisions due to insufficient driving skills and risk-taking tendencies such as speeding and reckless driving [8]. On the other hand, the primary causes of traffic collisions among older drivers include driver errors at intersections, failure to yield the right of way or heed stop signs or signals, unseen objects, etc. Visual and cognitive impairment due to aging are also associated with the higher risk of crash involvement, coupled with slower responses by older drivers [9, 10]. Furthermore, if older drivers suffer from chronic medical conditions, they are more likely to be involved in at-fault traffic collisions, indicating that the chronic condition itself could be a risk factor for traffic collisions [11]. However, Rolison et al. reported that the analysis of causative factors in traffic collision based on existing accident record may result in potential underreporting of some contributing factors such as driver distraction, drug and alcohol impairment, and uncorrected or defective eyesight [12]. Since policymakers rely on road accident statistics when implementing the new policy, a more accurate report regarding the causative factors of the accident should be provided. To improve the limitations of the current traffic collision reports, the authors proposed introduction of a system (e.g., use of mobile application) to enable completion of accident report form at the scene.

According to the traffic collision statistics of the Korea Road Traffic Authority, from 2000 to 2015, the total number of traffic collisions in South Korea declined from 290,481 (617.9 per 100,000) to 232,035 (458.4 per 100,000); however, the number of traffic collisions among older drivers aged 65 and older increased from 3,375 (62.2 per 100,000) to 23,063 (348.5 per 100,000) [13]. This dramatic increase in traffic collisions among older drivers has emerged as an important social issue in South Korea, but no previous studies have been conducted using a large-scale national database based on emergency departments (EDs) on the differences in characteristics between older and younger drivers. According to Bingham et al., when comparing traffic collision characteristics between adolescent and adult male and female drivers, adolescent male drivers were more likely to have single-vehicle and fatal head-on collision than adult male drivers, who had a higher likelihood of rear-end crashes [14]. Experience, driving style, or cognitive spatial abilities may reflect the differences in collision types. Similarly, single and multi-vehicle collisions may be different depending on the contributing factors to age difference [15]. We hypothesized that the collision type between the older and younger drivers can also show substantial distinction and believed clarifying these differences will be an important basis to establish policies for their traffic safety in the future. We also investigated the vehicle types because of its important role in characterizing traffic collision between driver groups, as the rate of serious injuries and fatalities according to vehicle types could vary [16].

This study aimed to identify the epidemiological characteristics and difference in the risk factors and types of traffic collision between older and younger drivers in South Korea.

## Materials and methods

### Study design and setting

This study was approved by the Korea Centers for Disease Control and Prevention (KCDC) and the institutional review board (IRB) of Kyungpook National University Hospital (IRB No. 2017-07-016-002), and informed consent was waived by the IRB because the data were analyzed anonymously. This retrospective, cross-sectional, observational study evaluated data of injured patients enrolled in the ED-based Injury In-depth Surveillance (EDIIS) system retrieved by the KCDC from January 2011 to December 2015. Since August 2006, the KCDC has been conducting EDIIS for all injured patients who visited EDs participating in the surveillance system to develop national polices for injury prevention. In 2006, the EDIIS system was initiated with a sample of 5 EDs; from 2010 to 2014, the number of participating EDs rose to 20. Twenty-three EDs across the nation have been participating in the surveillance system since 2015. The Ministry of Health and Welfare of Korea categorized EDs into three levels according to detailed criteria for facilities, resources, and personnel. During the study period, there were 20 level-1 emergency centers and 125 level-2 emergency centers; the emergency centers participating in the present study consisted of 10 level-1 emergency centers and 10 level-2 emergency centers in 2014, and three more level-2 centers were added in 2015.

### Study population

We included injured drivers aged ≥ 18 years who were registered in EDIIS during the study period. Included patients were limited to drivers of four or more wheels vehicles who were driving the vehicle and sustained an injury at the time of their collision. Among these, agricultural vehicles (including cultivators, tractors, and combines), industrial vehicles (such as bulldozers), and all-terrain vehicles were excluded. An ‘older driver’ was defined as a driver aged ≥ 65 years, according to the definition of a senior citizen established by the Road Traffic Act in South Korea.

### Data collection and variables

Data were collected by researchers at each hospital using the information from hospital electronic medical records based on the definition of EDIIS variables as guided by the KCDC guidebook. The collected data were entered into the website operated by the KCDC, which regularly conducted quality control activities on the collected data by providing feedback to each hospital on missing or incorrect variables, in order to improve data completeness.

We analyzed the following data extracted from the EDIIS database: patient demographics (sex, age), risk factors of injuries (season and times of injuries, alcohol consumption, use of seat belt, work-related injuries, types of vehicles and collisions), and outcomes of victims (ED disposition, hospital lengths of stay, and hospital deaths). Times of injury were classified into four categories; dawn (00:00 to 05:59), morning (06:00 to 11:59), afternoon (12:00 to 17:59), and night (18:00 to 23:59). Seat belt use was assessed based on statements from patients, their guardians, or paramedics who rescued the patients at the scene. Alcohol use was recorded based on statements of patients or their guardians because the EDs in South Korea do not routinely measure the blood alcohol levels of trauma patients. A work-related injury was defined as the activity of an injured patient recorded as ‘work’ at the time of injury. The vehicles were classified into the following types: vehicles with up to 10 seats (including motor cars, station wagons, minivans, jeeps, and sports utility vehicles), vehicles with 11–19 seats (minibuses, passenger vans, pick-up trucks, ambulances, and motorhomes), and vehicles with 20 or more seats (buses or coaches, trucks, and large/heavy transport vehicles), according to the KCDC guidelines. The types of collision were classified into single- and multi-vehicle collision based on the number of vehicles involved. Single-vehicle collision included only one vehicle, and multi-vehicle collision included two or more vehicles. Single-vehicle collision was further classified into three groups depending on the counterpart in the collision; ‘collision with fixed or stationary object’, ‘no counterpart’, and ‘others’. Fixed or stationary objects included parked vehicles, benches, trees, guard rails, and buildings. No counterpart was assigned if there was no collision. Examples of these include collision-free rollovers, sudden braking and swerving, going around a corner too quickly, vehicle collapse, and ejection from a vehicle. Hospital death was defined as death occurring within 30 days of the ED visit.

### Statistical analysis

All statistical analyses were performed using SAS software version 9.4 (SAS Institute, Inc., Cary, NC, USA). Linear trends in annual driver count from 2011 to 2015 were analyzed using Poisson regression, with simple linear regression being used in case of continuous data. Categorical data were represented as frequency and percentage, and linear trends in the proportion of each categorical variable from 2011 to 2015 were analyzed using Cochran-Mantel-Haenszel’s row means score difference test.

When comparing older and younger drivers, data were represented as the median and interquartile range (IQR; 25^th^ and 75^th^ percentiles), comparison of independent group means was done using Wilcoxon rank sum test because the data were found to be positively skewed in the Shapiro-Wilk test. Categorical data were represented as frequency and percentage, and comparison of proportions was done using Pearson’s chi-squared test. In all the analyses, *P* values < 0.05 were considered statistically significant.

## Results

**Fig 1:**
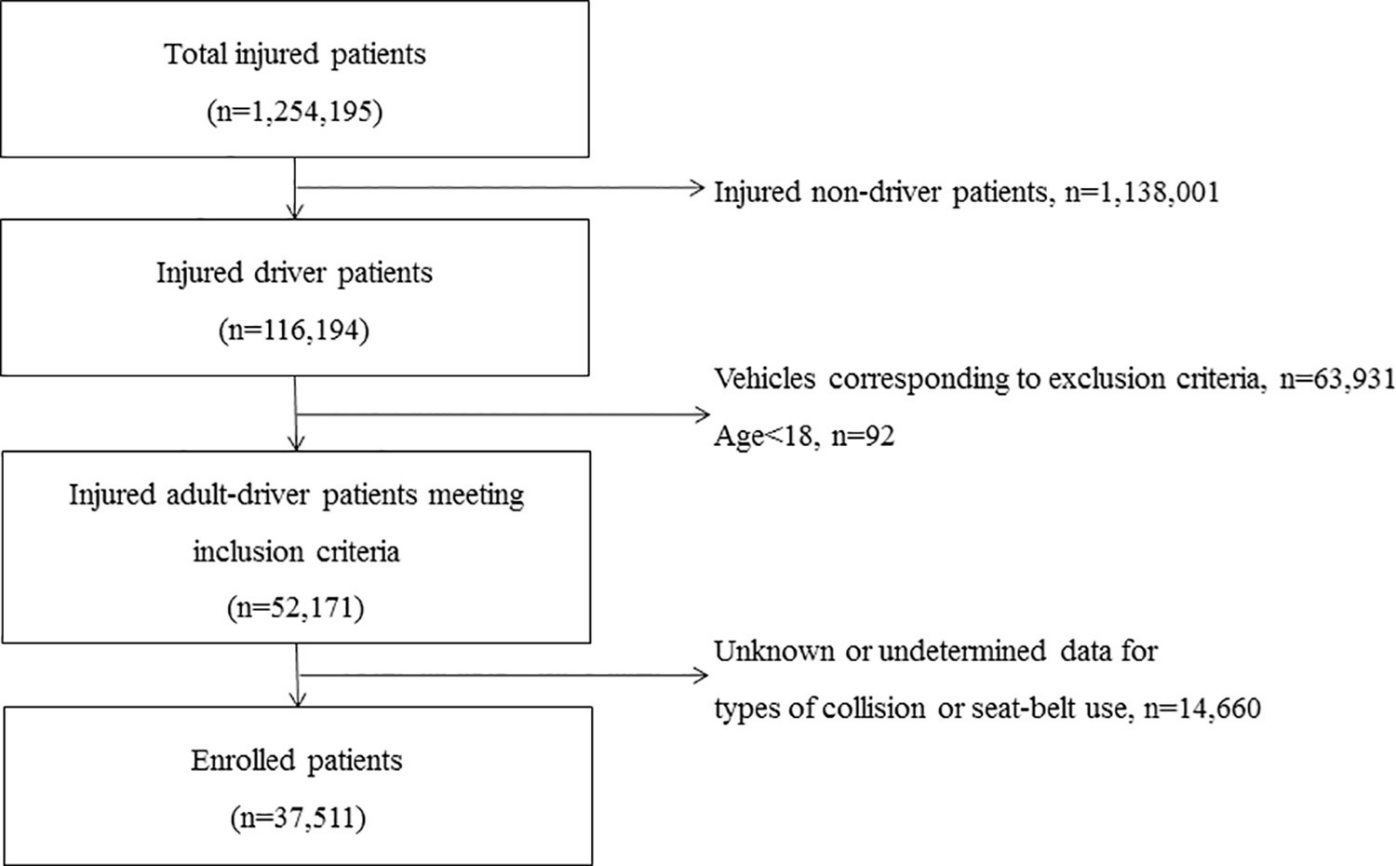
Study flow diagram of enrolled patients.

During the study period, there were 116,194 injured drivers registered in EDIIS and 52,171 met the inclusion criteria. An additional 14,660 were excluded for the following reason: unknown or undetermined data regarding types of collision and seat belt use. The final study population included 37,511 patients (Fig 1).

### Annual trends of epidemiological characteristics of traffic collision victims

The median age and IQR of all drivers were 41.0 (32.0–52.0), and 24,544 (65.4%) of them were men. The median age increased from 40.0 (IQR, 31.0–50.0) to 43.0 (IQR, 33.0–54.0) between 2010 and 2015 (*P* < 0.001). The proportion of older drivers increased annually from 5.0% in 2011 to 8.4% in 2015 (*P* < 0.001). Between 2010 and 2015, the fatality rate decreased from 3.1% to 1.2% (*P* = 0.287) for older drivers and from 0.9% to 0.5% (*P* = 0.009) for younger drivers. The annual trends of epidemiological characteristics of traffic collision victims are shown in Table 1.

**Table 1:** Annual trends of epidemiological characteristics of enrolled traffic collision victims.

	2011		2012		2013		2014		2015		*P* value
Driver numbers	6,460		8,104		7,713		8,073		7,161		<0.001 ^a^
Demographics											
Age ^b^	40 (31–50)		40 (31–51)		41 (31–52)		42 (32–53)		43 (33–54)		<0.001 ^b^
Age ≥ 65 years	323 (5.0%)		438 (5.4%)		455 (5.9%)		544 (6.7%)		601 (8.4%)		<0.001 ^c^
Sex, male	4,210 (65.2%)		5,382 (66.4%)		5,013 (65.0%)		5,295 (65.6%)		4,644 (64.9%)		0.358 ^c^
Season of injury											
Spring	1,428 (22.1%)		1,973 (24.3%)		1,790 (23.2%)		1,864 (23.1%)		1,793 (25.0%)		0.008 ^c^
Summer	1,625 (25.2%)		1,881 (23.2%)		1,952 (25.3%)		2,000 (24.8%)		1,666 (23.3%)		0.245 ^c^
Autumn	1,840 (28.5%)		2,056 (25.4%)		2,069 (26.8%)		2,182 (27.0%)		2,087 (29.1%)		0.038 ^c^
Winter	1,567 (24.3%)		2,194 (27.1%)		1,902 (24.7%)		2,027 (25.1%)		1,615 (22.6%)		<0.001 ^c^
Time of injury											
Morning	1,627 (25.2%)		2,043 (25.2%)		1,911 (24.8%)		2,002 (24.8%)		1,785 (24.9%)		0.558 ^c^
Afternoon	1,951 (30.2%)		2,457 (30.3%)		2,539 (32.9%)		2,554 (31.6%)		2,343 (32.7%)		<0.001 ^c^
Night	1,788 (27.7%)		2,209 (27.3%)		2,118 (27.5%)		2,242 (27.8%)		1,992 (27.8%)		0.597 ^c^
Dawn	1,076 (16.7%)		1,378 (17.0%)		1,133 (14.7%)		1,263 (15.6%)		1,028 (14.4%)		<0.001 ^c^
Unknown	18 (0.3%)		17 (0.2%)		12 (0.2%)		12 (0.1%)		13 (0.2%)		0.130 ^c^
Use of alcohol											
Yes	352 (5.4%)		439 (5.4%)		361 (4.7%)		354 (4.4%)		315 (4.4%)		<0.001 ^c^
No	5,882 (91.1%)		7,244 (89.4%)		6,828 (88.5%)		7,215 (89.4%)		6,225 (86.9%)		<0.001 ^c^
Unknown	226 (3.5%)		421 (5.2%)		524 (6.8%)		504 (6.2%)		621 (8.7%)		<0.001 ^c^
Seat belt use											<0.001 ^c^
Yes	5,537 (85.7%)		6,691 (82.6%)		6,442 (83.5%)		6,456 (80.0%)		5,920 (82.7%)		
No	923 (14.3%)		1,413 (17.4%)		1,271 (16.5%)		1,617 (20.0%)		1,241 (17.3%)		
Work-related injury	912 (14.1%)		860 (10.6%)		892 (11.6%)		949 (11.8%)		855 (11.9%)		0.029 ^c^
Types of vehicle											
Vehicles with up to 10 seats	6,172 (95.5%)		7,696 (95.0%)		7,309 (94.8%)		7,641 (94.6%)		6,747 (94.2%)		0.001 ^c^
Vehicles with 11–19 seats	108 (1.7%)		200 (2.5%)		199 (2.6%)		165 (2.0%)		118 (1.6%)		0.520 ^c^
Vehicles with 20 or more seats	180 (2.8%)		208 (2.6%)		205 (2.7%)		267 (3.3%)		296 (4.1%)		<0.001 ^c^
Types of collision											<0.001 ^c^
Multi-vehicle collision	5,088 (78.8%)		6,353 (78.4%)		6,074 (78.8%)		6,428 (79.6%)		5,804 (81.1%)		
Single-vehicle collision	1,372 (21.2%)		1,751 (21.6%)		1,639 (21.2%)		1,645 (20.4%)		1,357 (18.9%)		
ED disposition											
Discharge	5,022 (77.7%)		6,296 (77.7%)		6,073 (78.7%)		6,338 (78.5%)		5,695 (79.5%)		0.004 ^c^
Transfer	390 (6.0%)		468 (5.8%)		348 (4.5%)		418 (5.2%)		310 (4.3%)		<0.001 ^c^
Admission	1,002 (15.5%)		1,282 (15.8%)		1,242 (16.1%)		1,263 (15.6%)		1,110 (15.5%)		0.848 ^c^
Death	42 (0.7%)		50 (0.6%)		44 (0.6%)		40 (0.5%)		35 (0.5%)		0.111 ^c^
Unknown	4 (0.1%)		8 (0.1%)		6 (0.1%)		14 (0.2%)		11 (0.2%)		0.040 ^c^
Hospital LOS (minute) ^b^	139 (78–374)		134 (73–362)		125 (66–322)		127 (65–324)		115 (61–285)		<0.001 ^b^
Hospital LOS, discharged from ED (minute) ^b^	107 (68–192)		102 (65–184)		96 (58–171)		96 (58–179)		90 (55–162)		<0.001 ^b^
Hospital LOS, admitted from ED (minute) ^b^	17,254 (6,345–35,929)		15,928 (7,107–30,214)		14,844 (6,597–28,653)		12,596 (3,926–26,085)		12,041 (3,035–25,607)		<0.001 ^b^
Hospital death	64 (1.0%)		71 (0.9%)		61 (0.8%)		69 (0.9%)		37 (0.5%)		0.005 ^c^
Older driver	10 (3.1%)		9 (2.1%)		10 (2.2%)		7 (1.3%)		7 (1.2%)		0.287 ^c^
Younger driver	54 (0.9%)		62 (0.8%)		51 (0.7%)		62 (0.8%)		30 (0.5%)		0.009 ^c^

ED: emergency department; LOS: length of stay

^a^ *P* value is the test result of the linear trend using simple Poisson regression.

^b^ Data are represented as median and interquartile ranges. *P* value is the test result of the linear trend using simple linear regression.

^c^ *P* value is the test result of the linear trend using Cochran-Mantel-Haenszel’s row means score difference test.

### Comparison of risk factors between older and younger drivers

Of all the enrolled drivers, 2,361 (6.3%) were older drivers. The proportion of male older drivers was higher than that of male younger drivers (80.3% vs. 64.4%, *P* < 0.001). In older drivers, both seat belt use (79.0% vs. 83.0%, *P* < 0.001) and alcohol consumption (1.9% vs. 5.1%, *P* < 0.001) were lower than in younger drivers. According to the types of vehicle, the proportion of vehicles with 11–19 seats (3.0% vs. 2.0%) and vehicles with 20 or more seats (4.9% vs. 3.0%) were higher in older drivers than in younger drivers (*P* < 0.001). The risk factors of older and younger drivers are shown in Table 2.

**Table 2:** Comparison of risk factors between older and younger drivers.

	Total drivers (n = 37,511)		Older drivers (n = 2,361)		Younger driver (n = 35,150)		*P* value
Sex, male	24,544 (65.4%)		1,896 (80.3%)		22,648 (64.4%)		<0.001 ^a^
Age ^b^	41 (32–52)		69 (67–73)		40 (31–50)		<0.001 ^b^
Time of injury							<0.001 ^a^
Morning	9,368 (25.0%)		737 (31.2%)		8,631 (24.6%)		
Afternoon	11,844 (31.6%)		986 (41.8%)		10,858 (30.9%)		
Night	10,349 (27.6%)		406 (17.2%)		9,943 (28.3%)		
Dawn	5,878 (15.7%)		227 (9.6%)		5,651 (16.1%)		
Unknown	72 (0.2%)		5 (0.2%)		67 (0.2%)		
Use of alcohol							<0.001 ^a^
Yes	1,821 (4.9%)		45 (1.9%)		1,176 (5.1%)		
No	33,394 (89.0%)		2,169 (91.9%)		31,225 (88.8%)		
Unknown	2,296 (6.1%)		147 (6.2%)		2,149 (6.1%)		
Seat belt use							<0.001 ^a^
Yes	31,046 (82.8%)		1,865 (79.0%)		29,181 (83.0%)		
No	6,465 (17.2%)		496 (21.0%)		5,969 (17.0%)		
Types of vehicle							<0.001 ^a^
Vehicles with up to 10 seats	35,565 (94.8%)		2,174 (92.1%)		33,391 (95.0%)		
Vehicles with 11–19 seats	790 (2.1%)		72 (3.0%)		718 (2.0%)		
Vehicles with 20 or more seats	1,156 (3.1%)		115 (4.9%)		1,041 (3.0%)		

^a^ *P* value is the test result of the independent two group proportion difference using Pearson’s chi-squared test.

^b^ Data are shown as median and interquartile ranges. *P* value is the test result of the independent two group mean difference using Wilcoxon rank sum test.

There was a significant difference in seat belt use between older and younger drivers for females, for vehicles with up to 10 seats, and for vehicles with 20 or more seats (*P* < 0.05; Table 3).

**Table 3:** Comparison of seat belt use between older and younger drivers according to sex and types of vehicle.

	Seat belt use			*P* value
	Total drivers	Older drivers	Younger drivers	
Sex				
Male, %	80.6 (19,792/24,544) ^a^	79.0 (1,498/1,896) ^a^	80.8 (18,294/22,648) ^a^	0.061 ^b^
Female, %	86.8 (11,254/12,967) ^a^	78.9 (367/465) ^a^	87.1 (10.887/12,502) ^a^	<0.001 ^b^
Types of vehicle				
vehicle with up to 10 seats, %	83.3 (29,620/35,565) ^a^	80.2 (1,743/2,174) ^a^	83.5 (27,877/33,391) ^a^	<0.001 ^b^
vehicle with 11–19 seats, %	76.7 (606/790) ^a^	75.0 (54/72) ^a^	76.9 (552/718) ^a^	0.719 ^b^
vehicle with 20 or more seats, %	70.9 (820/1,156) ^a^	59.1 (68/115) ^a^	72.2 (752/1,041) ^a^	0.003 ^b^

^a^ Values in parentheses indicate number of drivers who used seat belt out of all drivers.

^b^ *P* value is the test result of the independent two group proportion difference using Pearson’s chi-squared test.

### Comparison of the types of collision between older and younger drivers and by age

**Fig 2:**
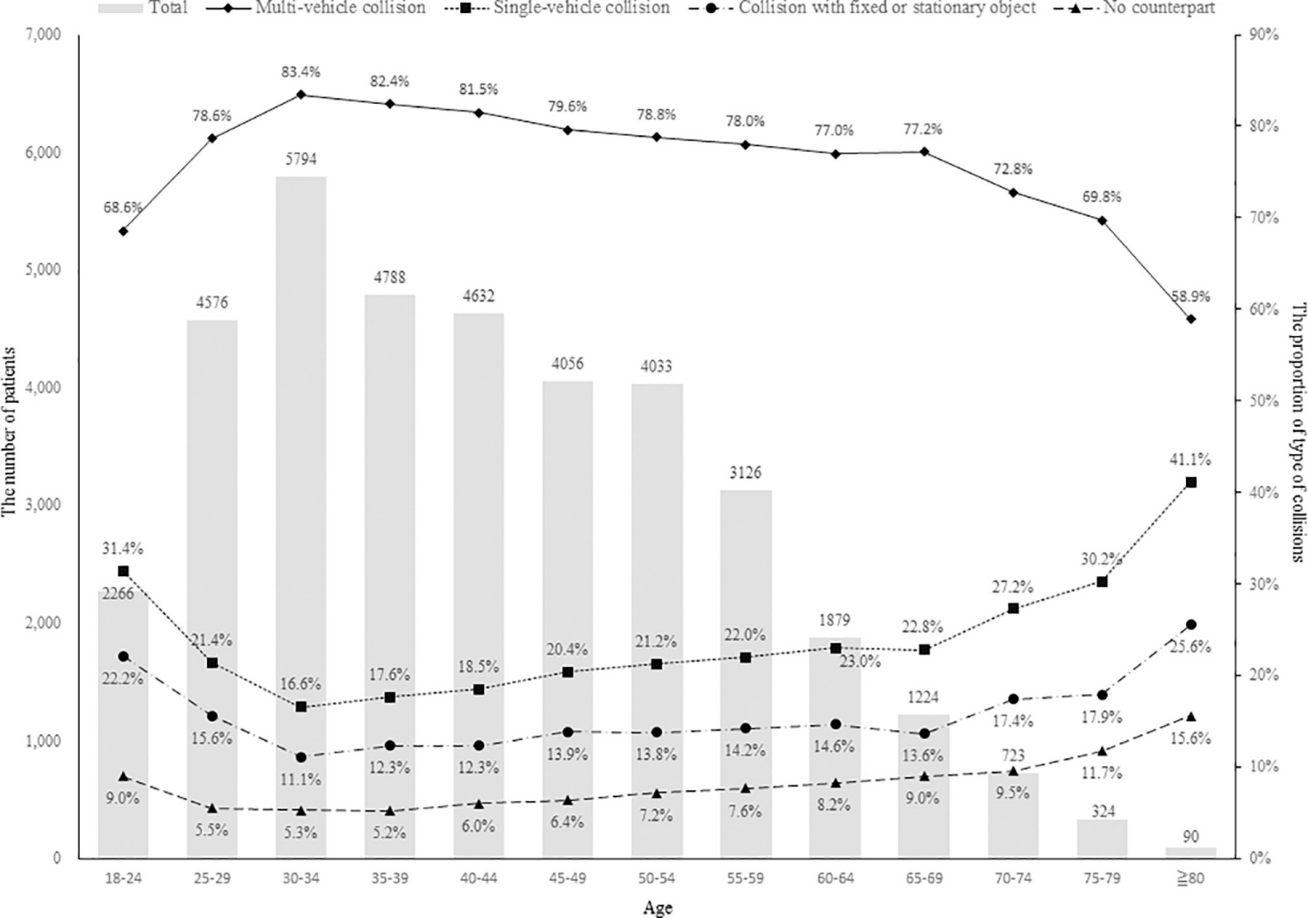
Distribution of the types of collision by age group.

The proportion of single-vehicle collision (25.9% vs. 20.3%) was higher in older drivers than in younger driver (*P* < 0.001; Table 4). Among the single-vehicle collision, older drivers had both higher proportion of ‘collision with fixed or stationary object’ (15.8% vs. 13.8%) and ‘no counterpart’ (9.8% vs. 6.4%) than in younger drivers, respectively. The proportions of multi-vehicle collision decreased sharply since the age of 70. The proportion of single-vehicle collision in drivers aged ≥ 80 years (41.1%) was the highest of all the age groups (Fig 2).

**Table 4:** Comparison of the types of collision between older and younger drivers.

	Total drivers (n = 37,511)	Older drivers (n = 2,361)	Younger driver (n = 35,150)	*P* value
Types of collision				<0.001 ^a^
Multi-vehicle collision	29,747 (79.3%)	1,750 (74.1%)	27,997 (79.7%)	
Single-vehicle collision	7,764 (20.7%)	611 (25.9%)	7,153 (20.3%)	
Collision with fixed or stationary object ^b^	5,232 (13.9%)	374 (15.8%)	4,858 (13.8%)	
No counterpart ^b^	2,468 (6.6%)	231 (9.8%)	2,237 (6.4%)	
Others ^b^	64 (0.2%)	6 (0.3%)	58 (0.2%)	

^a^ *P* value is the test result of the independent two group proportion difference using Pearson’s chi-squared test.

^b^ These are subgroups of the single-vehicle collision and not included in the analysis.

### Comparison of hospital outcomes between older and younger drivers

The proportions of hospital admissions from the ED in older drivers were significantly greater than those of younger drivers (25.9% vs. 15.0%, *P* < 0.001). The number of hospital deaths was 302, the older drivers had higher fatalities rate than younger drivers (1.8% vs. 0.7%, *P* < 0.001). The median hospital length of stay for older drivers was longer than that for younger drivers (206 min vs. 125 min; *P* < 0.001). The hospital outcomes of older and younger drivers are shown in Table 5.

**Table 5:** Comparison of hospital outcomes between older and younger drivers.

	Total drivers (n = 37,511)	Older drivers (n = 2,361)	Younger driver (n = 35,150)	*P* value
ED disposition				<0.001 ^a^
Discharge	29,424 (78.4%)	1,543 (65.4%)	27,881 (79.3%)	
Transfer	1,934 (5.2%)	170 (7.2%)	1,764 (5.0%)	
Admission	5,899 (15.7%)	612 (25.9%)	5,287 (15.0%)	
Death	211 (0.6%)	34 (1.4%)	177 (0.5%)	
Unknown	44 (0.1%)	2 (0.1%)	42 (0.1%)	
Hospital LOS (minute) ^b^	128 (69–331)	206 (94–842)	125 (67–315)	<0.001 ^b^
Hospital LOS, discharged form ED (minute) ^b^	98 (60–177)	129 (74–232)	97 (60–174)	<0.001 ^b^
Hospital LOS, admitted from ED (minute) ^b^	14,438 (5.443–28,738)	15,118 (4,315–32,444)	14,401 (5,531–28,468)	0.947 ^b^
Hospital death	302 (0.8%)	43 (1.8%)	259 (0.7%)	<0.001 ^a^

ED = Emergency department, LOS = Length of stay

^a^ *P* value is the test result of the independent two group proportion difference using Pearson’s chi-squared test.

^b^ Data are shown as median and interquartile ranges. *P* value is the test result of the independent two group mean difference using Wilcoxon rank sum test.

## Discussion

The current study found that the proportion of older drivers who visited the ED due to involvement in traffic collisions increased annually. This finding is likely associated with the growing elderly population and the proportions of elderly people possessing a driving license and driving on the road for travel or social activity. However, the fatality rate of older drivers declined over the same period, which is similar to previous studies. Cheung and McCartt [17] reported that the risk for fatal crashes among older drivers in the United States has declined between 1997 and 2008. Another study reported that the fatal crash involvement rate for drivers of all ages has declined significantly, and that this decline was greater for older drivers [18]. Consistent with these results, Hung et al. [19] reported that an increase in the proportion of healthy older adults would increase the likelihood of survival after a traffic collision. Advancement and popularization of vehicle safety technology, expanded road infrastructure, improvements in emergency medical services, and advanced trauma care can also contribute to reducing traffic collision-related mortality [20, 21]. The results of the present study show decreased annual fatality rate in both older and younger drivers; the decline was greater in older drivers. It is likely that the factors associated with this decline contributed positively to both older and younger drivers, but more effectively for older drivers.

Notably, we observed a significant difference in the proportion of single-vehicle collision between older and younger drivers. In a single-vehicle collision, responsibility for the collision is confined only to the driver and it is more likely to be associated with a driver’s driving ability. Therefore, the decline in coping ability due to aging in a collision situation in older drivers may contribute to the difference in the types of collision observed between the two groups. McGwin and Brown [8] reported that perceptual problems, such as judging and responding to traffic flow, were the most important differences between older and younger drivers. Physical, perceptual, and cognitive deficits associated with aging can negatively affect elders’ driving abilities; thus, they are more likely to be fatally injured on the road [10, 22–25]. On the other hand, some studies have reported that older drivers are aware of the changes in driving ability due to aging and can self-regulate [26, 27]. However, self-regulation regarding driving is only possible when the drivers are aware of the lack of their ability to drive. Considering the results of our study, it is possible that some older drivers who perceive themselves as healthy may not be able to respond as appropriately as younger drivers in a traffic collision.

To reduce the number of traffic collisions of older drivers, a variety of policies have been proposed. In Japan, the government has implemented a program for the voluntary return of driver’s licenses by older drivers and offers certain compensation to encourage voluntary participation [28]. However, most older drivers reported that they want to continue driving a private car instead of using public transportation, because they consider trains, subways, and buses to be inconvenient and misaligned with their travel needs, and taxis to be unsafe and too expensive [29]. In both rural and suburban areas, there is a lack of good public transportation options for the elderly even if they want to use them. To increase the voluntary return of older drivers’ licenses and to maintain the mobility of older drivers who decide to stop driving, they should be provided with safe, usable, and affordable alternative transportation options to replace the use of private vehicles and overcome the limitations of public transportation systems. Recently, commercial ridesharing systems such as Uber and Lyft have emerged as alternative methods of transportation that better meet the needs of the elderly population [30]. However, this is a relatively new system to South Korea, and thus it is not a popular or familiar option among the elderly; moreover, it is currently illegal in South Korea. We thus suggest that elderly people who have stopped driving be provided with government-subsidized door-to-door taxi services. Public transportation infrastructure should also be improved to make this a more accessible option for the elderly, both in rural and suburban areas.

In-person license renewal in the United States was associated with significantly lower fatality rates among older drivers since this process can provide a direct opportunity to identify potentially unsafe older drivers [31]. In New Zealand, drivers at age 75, 80, and every two years, should provide a medical certificate from a doctor indicating they are medically fit to drive before renewing a driving license, and some of them should pass on-road safety test or require confirmation from a specialist [32]. In Japan, a driving lesson became mandatory to drivers aged ≥ 70 years for license renewal since 2002, and all drivers aged ≥ 75 years have been obligated to take a cognitive functioning test since 2009 [33]. In South Korea in 2018, the government revised the Road Traffic Act, which includes shortening the license renewal interval from 5 to 3 years, and mandatory traffic safety lessons for older drivers aged ≥ 75 years. The traffic safety lesion consisted of a 2-h lecture including the cognitive test. However, any physical disability that can cause driving difficulties cannot be determined as older drivers are not required to provide a medical certificate when renewing their driver’s license. As the on-road driving test has also not been conducted at license renewal, assessing the objective driving ability of older drivers is currently limited. With this, many countries decided to shorten license renewal interval or have required mandatory safety education before license renewal for road safety in older drivers. However, deciding whether to restrict or stop driving based solely on older chronological age is also unreasonable, because the potential risks involved in driving vary even within the same older age group. Therefore, the policy to restrict or stop older people from driving should be carefully conducted in conjunction with an on-road driving test to evaluate their actual driving ability. However, conducting on-road driving tests for all licensed older drivers is impractical; thus, an alternative method is needed. Lee et al. reported that the results of driving-performance assessments conducted using simulators correlate highly with the results from conventional road tests [34]. Previous studies that have examined the relationship between the results of neurocognitive tests and fitness to drive among older adults have also demonstrated high correlations between these factors. The Mini-Mental State Examination, which is a cognitive screening test, has been used as predictor of driving ability [35]. The paper-based Trail Marker Test and computer-based Useful Field of View test measuring visual-cognitive functioning have been associated with the results of on-road test outcomes and prediction of future crash involvement. They could thus serve as useful screening tests and alternatives to on-road tests [36, 37]. However, since the results of single measurements predicting the results of on-road tests vary, further studies on the best predicting and most cost-effective and time-efficient tests for older adults’ driving capabilities are needed.

In our study, the temporal characteristics of the older drivers involved in motor vehicle crashes showed results similar to those of previous studies in which fewer traffic collisions were caused by older drivers at dawn and night-time than were caused by younger drivers [8]. This finding seems to be related to their driving tendency to avoid unsafe driving hours in addition to the lifestyle patterns of elderly with little outside activity at dawn and night. However, the rate of older drivers using seat belts was relatively lower compared with younger drivers, which is unlike a previous study [38]. The results of the present study showed that the larger the vehicle, the lower the seat belt use rate; a high proportion of large vehicles in older drivers may have influenced the lower seat belt use rate. In a previous study, the rate of drivers who responded as “always” wearing a seat belt was lower when driving commercial motor vehicles (such as truck, bus, or van) as compared to personal vehicles in the same drivers [39]. The reasons for not using a seat belt while driving a large vehicle were the feeling of safety due to the larger vehicle size and less police enforcement. Cock et al. reported that truck drivers had a 64% seat belt wear rate, which was approximately 20% lower than the national average rate in the United States [40]. Charbotel et al. reported that truck drivers were more likely to have 1.87 times higher Injury Severity Score ≥ 9 than that of car drivers, the low rate of seat belt use of truck drivers (14% of truck drivers vs. 72% of car drivers) could be a factor in explaining the difference of injury severity compared with car drivers [41]. We believe that a low perception of the need for safety observed in the drivers of large vehicles have resulted in low seat belt use in both older and younger drivers. However, even for small vehicle (<10 seater), the seat belt use rate of older drivers was lower than younger drivers. It was considered that factors related to the drivers themselves, besides the types of vehicles, also influenced the seat belt use rates. The results of a previous study showed that the majority of older drivers have confidence in driving except in specific difficult driving situations and they tend to evaluate their driving ability as not bad [42]. This seems to be the result of their long duration of driving experience or the tendency to ignore physical changes due to aging. The perception that fewer fatal crashes will occur due to the tendency of older drivers to avoid long-distance driving and to drive only to familiar places may also have affected the results of the present study. However, it is already known that self-rated driving confidence and on-road driving performance among older drivers have little association [43]. In the future, it will be necessary to strengthen police enforcement for unfastened seat belts for all drivers of large vehicles, and education and public relations related to the degradation of driving performance and the importance of seat belt use for older drivers.

We found that the proportion of older drivers has increased in the present study, but we could not explore whether collision rates of older drivers have also increased during the same period because our data only included traffic collision patients who visited the EDs of participating hospitals. Previous study has reported that failure control for drivers of all ages involved in collisions overestimated the collision risks of older drivers [44]. Although there is a lack of consideration of traffic collision rates, the authors believe that the current study’s findings of a higher proportion of single-vehicle collisions with driver responsibility and a lower seat belt use rate among older drivers compared to younger drivers are meaningful because they provide important evidence that road safety interventions for older drivers should be emphasized in the future.

This study has several limitations. First, not all hospitals in South Korea participated in the study and the hospitals in the study consisted mainly of relatively high-level emergency centers located in urban areas. There is thus a possibility of selection bias in the present study. Second, we could not consider the following information that might affect age differences in collision counts and the cause of traffic collisions, because EDIIS did not collect it: vehicle speed, the period for which the driving license was held, weather, roadway environment, annual mileage and number of trips. Rolison et al. reported that the driving risk of older drivers did not greatly increase when assessed based on the travel pattern incorporating the annual mileage, travel frequency, and travel duration, not the annual mileage alone [45]. This result suggests that the driving risk assessment have to be interpreted based on the incorporated results of multiple components rather than a single component for risk exposure. Third, the number of EDs that participated in the study increased during the study period (20 hospitals in 2011–2014, 23 hospitals in 2015). This may have affected the results of this study, but only minimally. Finally, we considered that single-vehicle collision might be associated with decreased driving performance. However, this type of collision can also occur irrespective of an individual’s driving ability, for example, in the cases of careless driving, and road or vehicle problems. Although there may be insufficient consideration of these points, we believe that the results of this study are meaningful since the possible occurrence of unrecorded variables could apply equally to both older and younger drivers.

## Conclusions

We found that the proportion of older drivers increased annually, and older drivers experienced more single-vehicle collision and used seat belts less frequently than younger drivers. Based on these results, national policy support to reduce traffic collision by older drivers is required, and public relation activities and traffic enforcement should be strengthened to increase the seat belt use rate for older drivers.
